# Iminosugars counteract the downregulation of the interferon γ receptor by dengue virus

**DOI:** 10.1016/j.antiviral.2019.104551

**Published:** 2019-10

**Authors:** Joanna L. Miller, Michelle L. Hill, Juliane Brun, Andrew Pountain, Andrew C. Sayce, Nicole Zitzmann

**Affiliations:** Oxford Glycobiology Institute, Department of Biochemistry, University of Oxford, Oxford, United Kingdom

**Keywords:** Dengue virus, Macrophage, Iminosugar, Antiviral, Receptor, Mannose receptor, Interferon gamma receptor

## Abstract

The antiviral mechanism of action of iminosugars against many enveloped viruses is hypothesized to be a consequence of misfolding of viral N-linked glycoproteins through inhibition of host endoplasmic reticulum α-glucosidase enzymes. Iminosugar treatment of dengue virus (DENV) infection results in reduced secretion of virions and hence lower viral titres *in vitro* and *in vivo*. We investigated whether iminosugars might also affect host receptors important in DENV attachment and uptake and immune responses to DENV.

Using a primary human macrophage model of DENV infection, we investigated the effects of maturation with IL-4, DENV-infection and treatment with *N*-butyl-1-deoxynojirimycin (*N*B-DNJ) or *N*-(9-methoxynonyl)-1-DNJ (M*O*N-DNJ) on expression of 11 macrophage receptors.

Whereas iminosugars did not affect surface expression of any of the receptors examined, DENV infection significantly reduced surface IFNγ receptor amongst other changes to total receptor expression. This effect required infectious DENV and was reversed by iminosugar treatment. Treatment also affected signalling of the IFNγ receptor and TNFα receptor. In addition, iminosugars reduced ligand binding to the carbohydrate receptor-binding domain of the mannose receptor. This work demonstrates that iminosugar treatment of primary macrophages affects expression and functionality of some key glycosylated host immune receptors important in the dengue life cycle.

## Highlights

• 11 macrophage receptors were screened for expression following DENV infection and iminosugar treatment.
• Iminosugar treatment alone resulted in only modest intracellular accumulation of the IFNγ and TNFα receptors.
• DENV infection decreased surface and total levels of the IFNγ receptor.
• Iminosugar treatment reversed the DENV mediated down-regulation of total expression of the IFNγ receptor.
• Iminosugar treatment affected signalling of the IFNγ receptor and TNFα receptor.
• Iminosugar treatment affected ligand binding to the carbohydrate receptor-binding domain of the mannose receptor.

Iminosugars are glucose mimetics with a nitrogen replacing the ring oxygen, that hold promise for the development of broad-spectrum antivirals. Two compounds, *N*-(9-methoxynonyl)-1-deoxynojirimycin (M*O*N-DNJ, also called UV-4) and celgosivir have been included in DENV clinical trials (NCT01619969, NCT02569827 and NCT02061358). The major mechanism of iminosugar antiviral activity is believed to be misfolding of viral N-linked glycoproteins through the competitive inhibition of host endoplasmic reticulum (ER) α-glucosidases I and II (Miller et al., 2018) and treatment with iminosugars leads to reduced secretion and infectivity of dengue virions (Sayce et al., 2010, 2016). Iminosugar-induced viral glycoprotein misfolding may not be the only antiviral mechanism of these compounds. Inhibiting the glycoprotein folding quality control mechanisms in the ER may also result in changes to host proteins. Many host proteins central for DENV attachment, uptake, signalling and the immune response are themselves N-linked glycoproteins and thus perturbations in their expression, glycosylation and function could have implications for virus growth. Here we investigated whether iminosugar treatment leads to changes in the expression, cellular distribution and functionality of key host-cell receptors as part of the antiviral effect.

Human monocyte-derived macrophages (MDMΦ) model a key target cell of DENV; however, they are relatively refractory to infection by DENV *in vitro*. Using an established model system in which MDMΦ susceptibility to DENV infection is enhanced by pre-treatment with IL-4 (Miller et al., 2008) we evaluated the effects of IL-4 treatment, DENV infection, and iminosugar treatment on the expression and function of important macrophage-expressed receptors. The receptors selected for analysis play key roles in the recognition, binding, entry and immune response to DENV infection as listed in Supplemental Table 1.

We used flow cytometry to compare the effects of DENV infection and iminosugar treatment on the expression of 11 MDMΦ receptors. Human peripheral blood mononuclear cells were isolated from buffy coats (NHS Blood and Transport) by centrifugation over a Ficoll-Paque™ PLUS (Amersham) gradient and monocytes isolated by adherence as previously reported (Miller et al., 2008). Autologous plasma was collected, heat-inactivated (56 °C, 30 min), and used to supplement (1%) X–VIVO10 (Lonza) medium to produce MDMΦ growth medium (MGM). Monocytes were differentiated for 3 days (37 °C, 5% CO_2_) in MGM with or without 25 ng/ml recombinant human IL-4 (PeproTech) to generate partially matured MDMΦs, then infected with DENV2 strain 16681 (a gift from G. Screaton, Oxford, UK) diluted to a multiplicity of infection (MOI) of 1 in X–VIVO10 without supplements for 90 min, followed by treatment with no iminosugar, 100 μM *N*B-DNJ (solubilised in PBS, gift from Oxford GlycoSciences Ltd.), or 25 μM M*O*N-DNJ (solubilised in acidified water, gift from United Therapeutics) for 2 days. These concentrations of iminosugars are greater than those required to inhibit 90% of release of infectious virus from infected MDMΦ, are >125-fold lower than the 50% cytotoxicity concentrations (Miller et al., 2012) and confirmed to contain less than 0.05 endotoxin units per ml. The use of human blood was approved by the NHS National Research Ethics Service (09/H0606/3). Total and surface expression levels for all receptors under all 12 treatment conditions are shown in Supplemental fig. 1. Many of the receptors examined were expressed at various levels on cells from individual donors (eg. surface CCR5), while other receptors show more uniform expression (eg. IFNγ receptor (IFNγR) and TNFα receptor (TNFαR)). A combination of examining the mean normalised gMFI (geometric mean fluorescence intensity) values (Supplemental fig. 1) and significance of the changes (Supplemental Tables 2 and 3) shows effects of IL-4 (upregulating surface MR, CD11b and CLEC5A, as well as total MR levels and down-regulating CD14), DENV (down-regulating surface and total IFNγR) and iminosugars (upregulation of total IFNγR and TNFαR). The effects of IL-4 have been reported previously (Chehimi et al., 2003; Deszo et al., 2004; Miller et al., 2008; Pickl et al., 1996; Steinbach and Thiele, 1994) though the opposite effect of IL-4 on CLEC5A was reported (Gonzalez-Dominguez et al., 2015). The differing degrees of variation in normalised gMFI values observed between donors for the expression of receptors, are likely the result of variance in both absolute receptor expression in the outbred human population, antibody avidity and variation in the percentage of cells infected when MDMΦ are infected with DENV at a MOI 1 (Cowley et al., 2012). The variation observed highlights the challenges and importance of data from primary human macrophages, in comparison to cell lines where data obtained may only be relevant to one particular genotype. The balance between the degree of effect (fold-change) and significance of the change, needs to be considered, however the most apposite measure of effect ultimately, is whether there is any functional outcome. Three receptors were chosen for investigation of functional effects following iminosugar treatment.

**Fig. 1:**
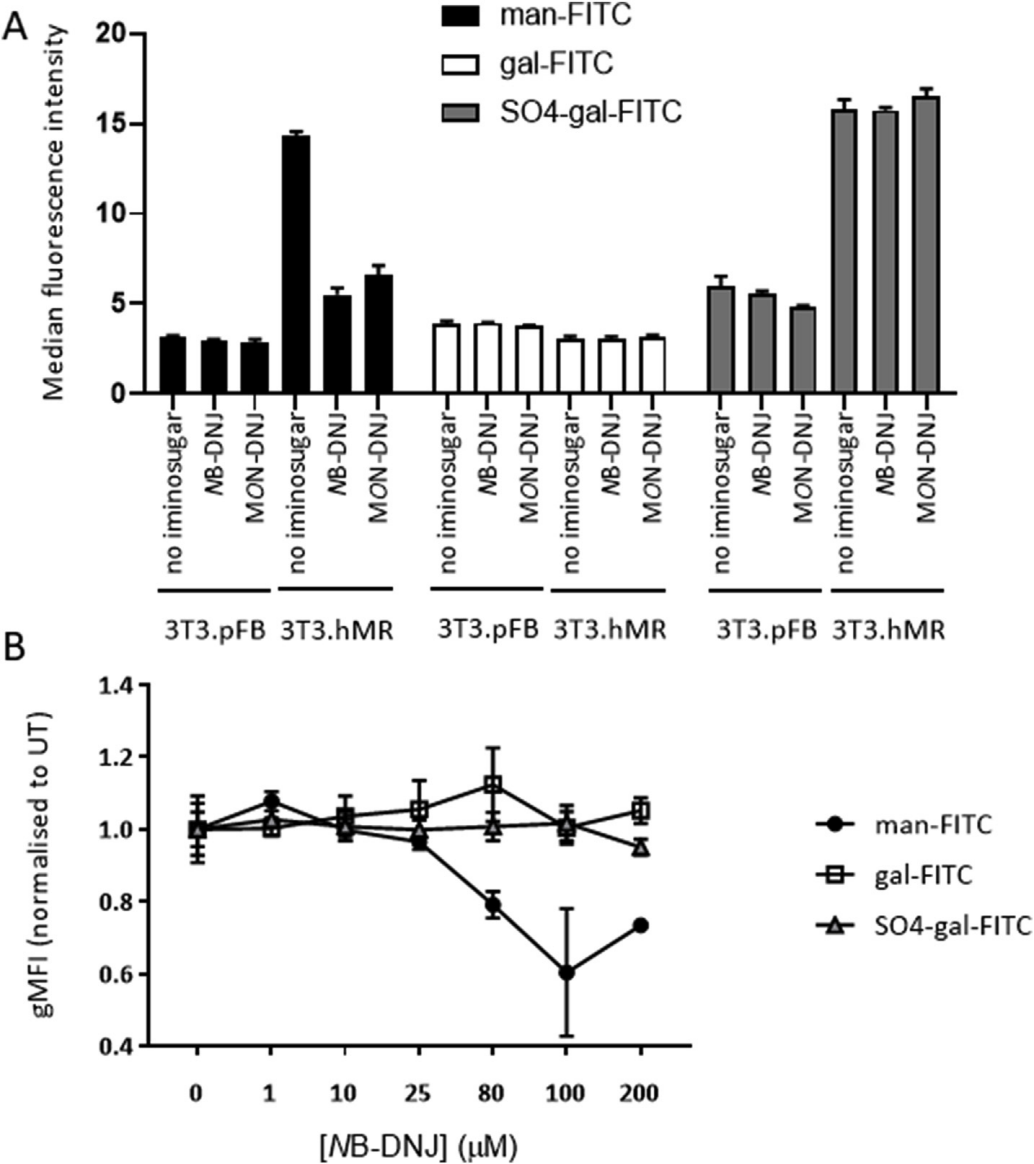
**Iminosugars specifically reduce MR association with a CRD ligand.** 3T3.hMR and 3T3.pFB were grown in the presence of *N*B-DNJ or M*O*N-DNJ, washed, then incubated for 2 h at 37 °C with 5 μg/ml FITC-labelled sugar ligands (α-D-mannose-PAA-FITC (man-FITC), α-D-galactose-PAA-FITC (gal-FITC) or β-D-galactose-3-sulfate-PAA-FITC (SO_4_-gal-FITC) (GlycoTec)). The mannosylated ligand, man-FITC, is a specific endocytic tracer for ligands of the carbohydrate recognition domains (CRD) of the MR which bind mannose, fucose, and *N*-acetylglucosamine (Pontow et al., 1992); the sulphated galactose ligand, SO_4_-gal-FITC, is specifically bound by the cysteine-rich domain of the MR; while the galactosylated ligand, gal-FITC, shows background binding levels as it is not bound specifically by any domains of the MR. This assay measured associated ligand which was either bound or taken up into the cells as unbound ligands were washed away before association of FITC-labelled sugar with the cell was assessed by flow cytometry. (A) Single representative experiment showing growth of 3T3.pFB and 3T3.hMR cells for 2 days with 100 μM NB-DNJ or 25 μM M*O*N-DNJ before assessing binding and uptake of FITC-sugar ligands, performed in triplicate. Background levels of association of each FITC-sugar to 3T3. pFB cells are shown. (B) Ligand binding to 3T3.hMR cells grown for 2 days in the presence of a titration of *N*B-DNJ, gMFI for each ligand normalised to levels bound in the absence of *N*B-DNJ, pooled data from 5 experiments, each performed in triplicate, mean±SD are shown.

To elucidate whether iminosugars have any effect on the function of the MR, NIH3T3 murine fibroblasts stably transfected with human MR (3T3. hMR) and control cells expressing the vector only (3T3. pFB) were used (Miller et al., 2008). This avoids complications due to MDMΦ expressing both the MR and DC-SIGN receptors which have overlapping ligand specificity. Two days culture in the presence of 100 μM NB-DNJ or 25 μM M*O*N-DNJ slightly (1.3-fold), but significantly, upregulated surface and total expression levels of MR on 3T3. hMR (Supplemental fig. 2). To assess whether growth in the presence of iminosugars altered MR function, we incubated MR transfectants grown in the presence or absence of iminosugars with FITC-labelled sugars for 2 h at 37 °C, washed away un-associated ligand then quantified ligand association by flow cytometry. We exploited the specificity of different domains of the MR for particular glycans to determine whether growth with iminosugars affected binding to the different lectin domains of the MR. 3T3. hMR cells bound both man-FITC and SO_4_-gal-FITC at 5-fold greater levels than non-specific association seen with gal-FITC (Fig. 1A). Growth of 3T3. hMR cells in the presence of iminosugars reduced association of man-FITC (a specific ligand of the carbohydrate recognition domains (CRD) of the MR), but not SO_4_-gal-FITC, by 2–2.6-fold, under the same conditions that slightly increased surface expression of the MR. This specific effect of *N*B-DNJ on functionality of the CRD of the MR showed a dose response (Fig. 1B).

**Fig. 2:**
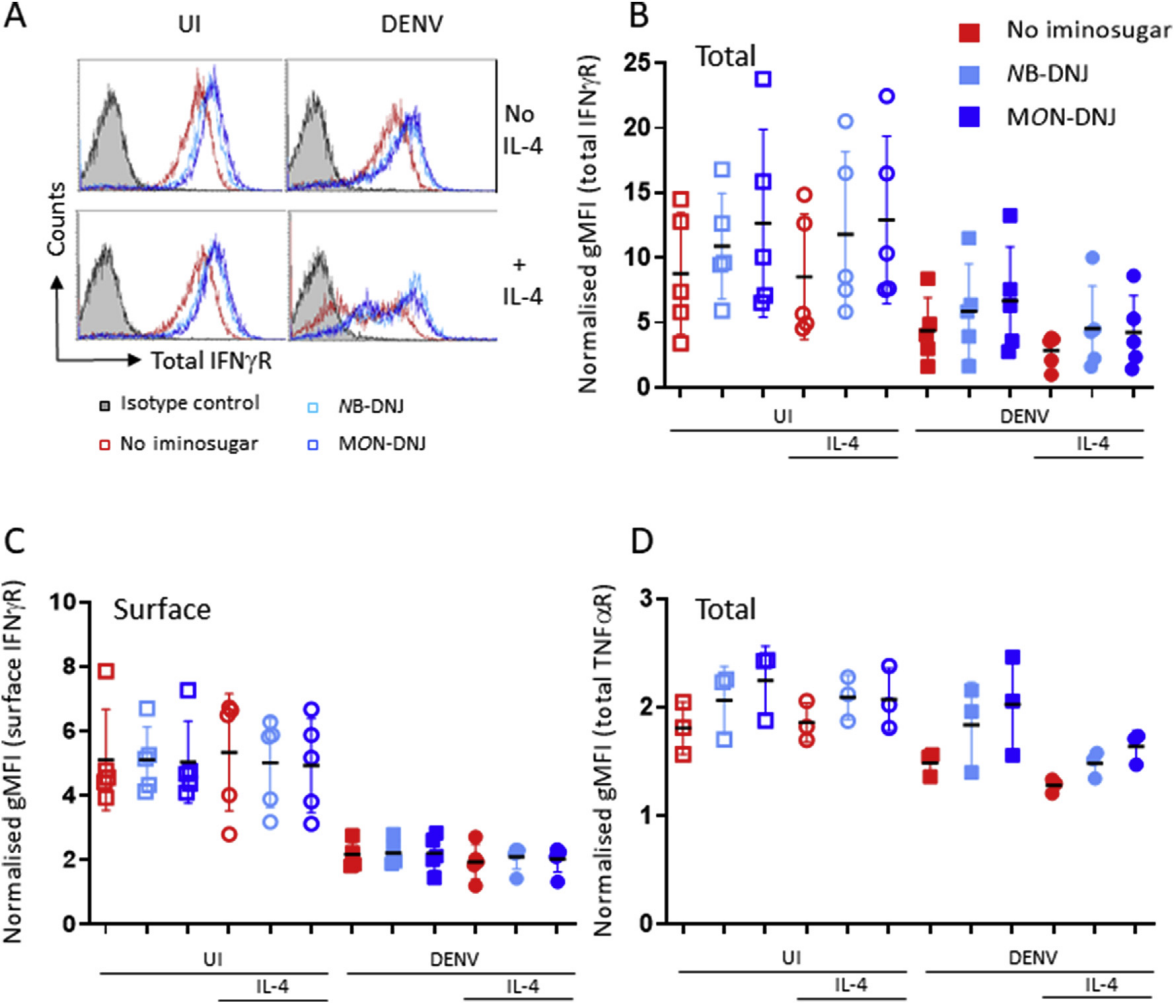
**Effects of IL-4 treatment, DENV infection and iminosugar treatment on IFNγ receptor and TNFα receptor expression.** Monocytes isolated from the blood of multiple donors were incubated for 3 days with or without 25 ng/ml rec human IL-4, before being infected with DENV2 at a MOI of 1 for 90 min or mock-treated. Media with or without virus was aspirated from the cells and replaced with media alone, or supplemented with 100 μM NB-DNJ or 25 μM M*O*N-DNJ. Following 2 days incubation, the cells were resuspended by scraping, fixed and stained as described in Supplemental Fig. 1 legend and analysed by flow cytometry for receptor expression. (A) Representative histogram plots of cells from a single donor for the total expression of IFNγR under the different treatments as specified in the legend. Grey filled histogram represents non-specific background as determined with cells stained with an IgG2a isotype control. The geometric mean fluorescent intensity (gMFI) of (B) total IFNγR, (C) surface IFNγR and (D) total TNFαR expression under the twelve conditions was normalised to isotype control staining. Normalised gMFI levels from cells from multiple donors are shown individually, as well as mean and SD.

**Fig. 3:**
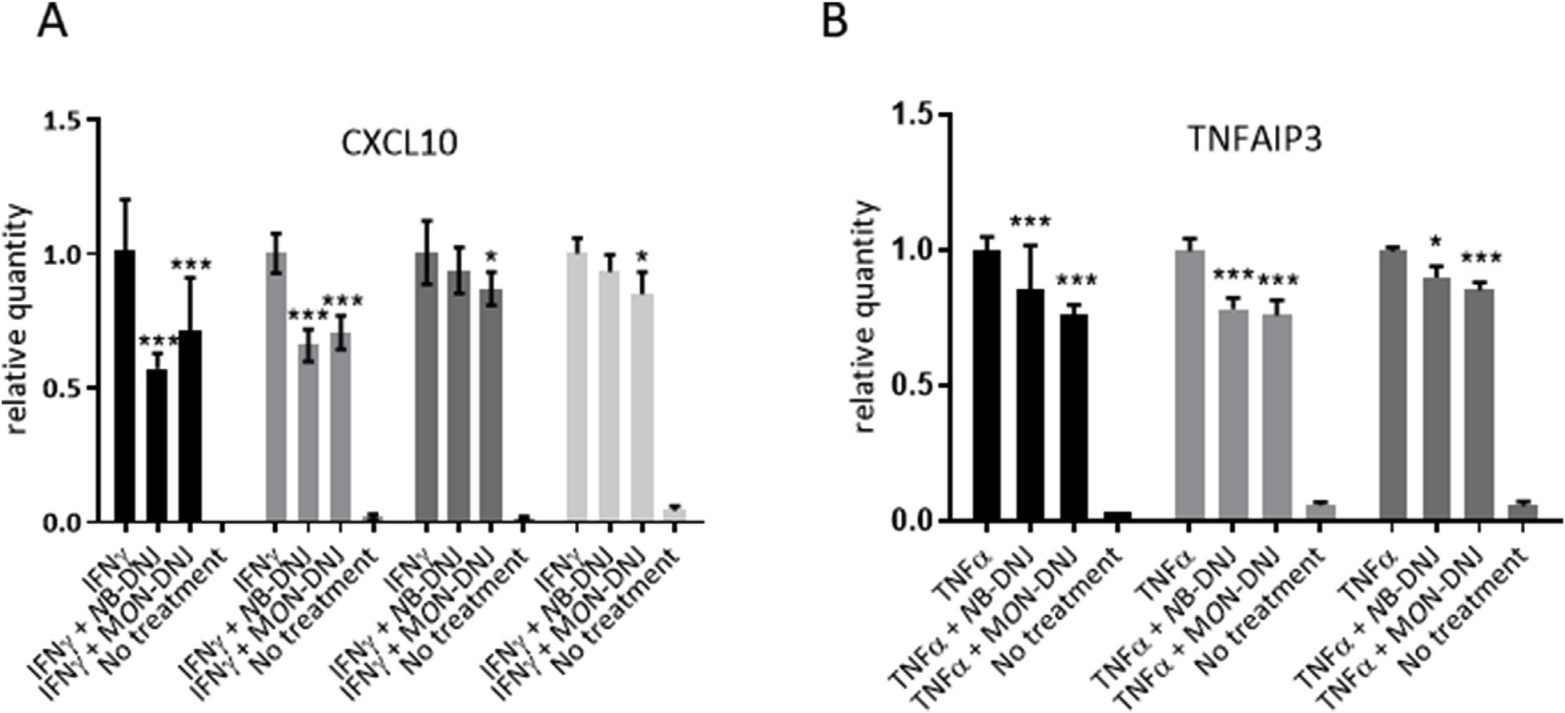
**Iminosugars dampen the response signalled by IFNγ receptor and TNFα receptor.** Primary human MDMΦs matured in the presence of IL-4 were treated with iminosugars for 2 days (100 μM NB-DNJ or 25 μM M*O*N-DNJ) then stimulated with either (A) 250 pg/ml recombinant human IFNγ (R&D Systems) or (B) 250 pg/ml TNFα (Peprotech), or mock stimulated (no treatment). RNA was extracted from the cells 6 h later (Direct-zol™ RNA Mini-prep kit (Zymo Research)) and RT-qPCR performed (using the Verso 1-step RT-qPCR kit in a volume of 20 μl according to the manufacturer's instructions on an Applied Biosystems 7500 real-time PCR system) for (A) CXCL10 (n = 4 donors cells) or (B) TNFAIP3 (n = 3 donors cells). TaqMan Gene Expression Assays containing gene-specific probe and primer sets were used for quantitative gene expression analysis of CXCL10 (assay ID Hs01124252_g1) and TNFAIP3 (assay ID Hs00234713_m1), ribosomal protein lateral stalk subunit P2 (RPLP2) (assay ID Hs01115128_gH) was also amplified and used as an endogenous control (all Thermo Fisher). 2 μl of each sample was analysed in technical duplicate and RNA quantified by the ΔΔCt method. The relative quantity of RNA is shown for individual donors, normalised to an RPLP2 endogenous control, mean and SD. A two-way ANOVA comparing between treatments using Holm-Šidák correction for multiple comparisons was performed and those treatments with significant changes in comparison with levels produced by cytokine stimulus in the absence of iminosugar are highlighted with asterisks. *p < 0.05, **p < 0.005, ***p < 0.0005.

Iminosugars significantly upregulated total IFNγR and TNFαR levels regardless of IL-4 or DENV-infection. Total levels of the IFNγR were affected not only by the presence of iminosugars, but also by DENV infection, as seen in shifts in histograms showing altered expression levels from an individual donor (Fig. 2A) and in normalised gMFI (Fig. 2B) data from 5 donors. While DENV infection resulted in reduced total (Fig. 2B and surface (Fig. 2C) expression of IFNγR, this was countered by iminosugars, which increased total receptor levels. A similar trend was observed for total expression of the TNFαR (Fig. 2D). The effect on the IFNγR was both titratable and required infectious virus. When decreasing MOIs of DENV were used to infect macrophages, even the lowest MOI tested (0.001) reduced the surface expression of the IFNγR two-fold (Supplemental Fig. 3A and 3B). The percentage of MDMΦ infected with DENV at MOI 0.001 is below the limit of detection as is release of infectious virus (data not shown). Treatment with UV-inactivated DENV did not down-regulate the IFNγR (Supplemental fig. 3C), implying that viral replication is required. Expression of IFNγR started to drop between 12 and 24 h following infection by DENV (Supplemental Fig. 3D and 3E). To evaluate whether iminosugar-induced changes in receptor expression levels had functional consequences on IFNγR and TNFαR signalling we performed RT-qPCR assays for mRNA levels of specific genes transcribed in response to either IFNγ or TNFα stimulation of MDMΦ. We selected a shortlist of genes for which the mRNA levels were not affected by iminosugar treatment (GEO file: GSE128303). From this list, CXCL10 and TNFAIP3 were selected for analysis, responding specifically to signalling induced via the IFNγR or TNFαR, respectively (data not shown). MDMΦ matured in the presence of IL-4 were treated for 2 days with iminosugars to allow for turnover of receptor in the presence of iminosugar, then treated for 6 h with either IFNγ or TNFα before RNA was isolated for quantification of CXCL10 and TNFAIP3, respectively. Culture in the presence of at least one of the two iminosugars examined significantly dampened the response signalled by both of these receptors. (Fig. 3).

This study shows that IL-4 and DENV infection affect the expression levels of some key DENV attachment/entry receptors on primary human macrophages. In contrast, iminosugars do not independently affect receptor expression but are able to counteract the downregulation of the IFNγR by DENV. Furthermore, iminosugars affect the function of some of these receptors. MR ligand binding to the CRD was reduced on cells grown in the presence of iminosugars, and iminosugars also modulated downstream signalling of two immune receptors important in the context of DENV infection, the IFNγR and the TNFαR. These findings demonstrate that important functional effects may be present in the absence of a change in the observed protein expression levels following iminosugar treatment.

Viruses modulate cell surface receptors, often for viral immune evasion and pathogenesis, and DENV is no exception. Our experiments showed that DENV infection of MDMΦ down-regulated intracellular pools of MR, and downregulated surface and total expression of the IFNγR and TNFαR. The down-regulation of the IFNγR and TNFαR may be a hitherto unknown mechanism by which DENV reduces macrophage responsiveness to IFNγ and TNFα. IFNγR surface expression is known to be down-regulated by a number of mechanisms, including type I IFNs (Bach et al., 1997; Schroder et al., 2004).

In contrast to DENV infection, treatment with iminosugars alone had no significant effects on either the surface or total expression levels of the attachment/entry receptors examined. Growth in the presence of either *N*B-DNJ or M*O*N-DNJ did however upregulate total levels of IFNγR (p = 0.110 and 0.050, respectively) and TNFαR (p = 0.013 and 0.002, respectively), without altering surface expression. This suggests accumulation of receptor intracellularly, possibly due to the rate of production of misfolded proteins overtaking the rate of their degradation. For TNFαR, which is expressed both on the cell surface and cleaved from the membrane resulting in soluble TNFαR, the rate of cleavage from the membrane may have overtaken the rate of *de novo* receptor production.

It was important to follow the analysis of receptor expression by determining whether iminosugars had any functional impacts on downstream pathways. The importance of signalling in response to IFNγ in DENV infection has been highlighted in murine studies where IFNγ receptor signalling is critical in resolving DENV infection (Shresta et al., 2004) and in restricting systemic virus infection and central nervous system disease (Prestwood et al., 2012). In addition, it is known that DENV inhibits the IFNγ and TNFα signalling pathways (Gack and Diamond, 2016). Iminosugars significantly dampen the induction of these mRNAs in response to the ligands for the IFNγ or TNFα receptors, although the degree of effect varies between individual donors.

N-glycosylation is known to affect functionality and ligand-binding affinity for several receptors examined here and it was therefore important to demonstrate whether functionality was affected, even when expression was not altered. Glycosylation does not substantially affect MR biosynthesis, proteolytic processing and trafficking (Su et al., 2005a), which is consistent with our observation that growth of 3T3. hMR transfectants or primary macrophages with iminosugars did not reduce the surface or total levels of MR. However, growth of the MR transfected cell line in the presence of >80 μM NB-DNJ or 25 μM M*O*N-DNJ for 2 days specifically reduced functionality of the CRD of the MR. Changes in sialylation (and carbohydrate sulphation) affect MR function (Martinez-Pomares, 2012). In particular, lack of terminal sialylation leads to a reduction of mannosylated ligand binding activity (Su et al., 2005a, 2005b) and the presence of neutral glycans (ie. less sialic acid) promotes formation of multimeric structures of MR and indirectly increases avidity for sulphated carbohydrate ligands. Changes such as these as a consequence of altered glycan processing due to inhibited interaction with the calnexin/calreticulin quality control mechanisms in the ER may explain the altered ligand binding of the CRD of the MR on cells grown in the presence of iminosugars.

Here we demonstrate that iminosugars affect expression levels and signalling of host receptors important in DENV infection and disease. Ongoing work will aim to establish whether there is a direct relationship between these findings and the anti-DENV activity of iminosugars. Does the iminosugar-induced upregulation of IFNγR or TNFαR contribute the enhanced antiviral state in macrophages? Does the iminosugar-induced misfolding of MR have an inhibitory effect on the entry process of DENV? Establishing the consequences of these receptor changes will lead to a better understanding of the multiple modes of antiviral action of this promising class of antiviral agents for the treatment of acute viral infections.

## Appendix A Supplementary data

Supplemental Tables 2 and 3 Supplemental Tables 2 and 3

Data Profile Data Profile
