# Supplementary material for: Iminosugars counteract the downregulation of the interferon γ receptor by dengue virus
Source: Antiviral Res. 2019 Oct;170:104551. doi: 10.1016/j.antiviral.2019.104551 (PMC6891261; doi:10.1016/j.antiviral.2019.104551)
Supplement: Supplemental Figures.pdf [file mmc1.pdf]

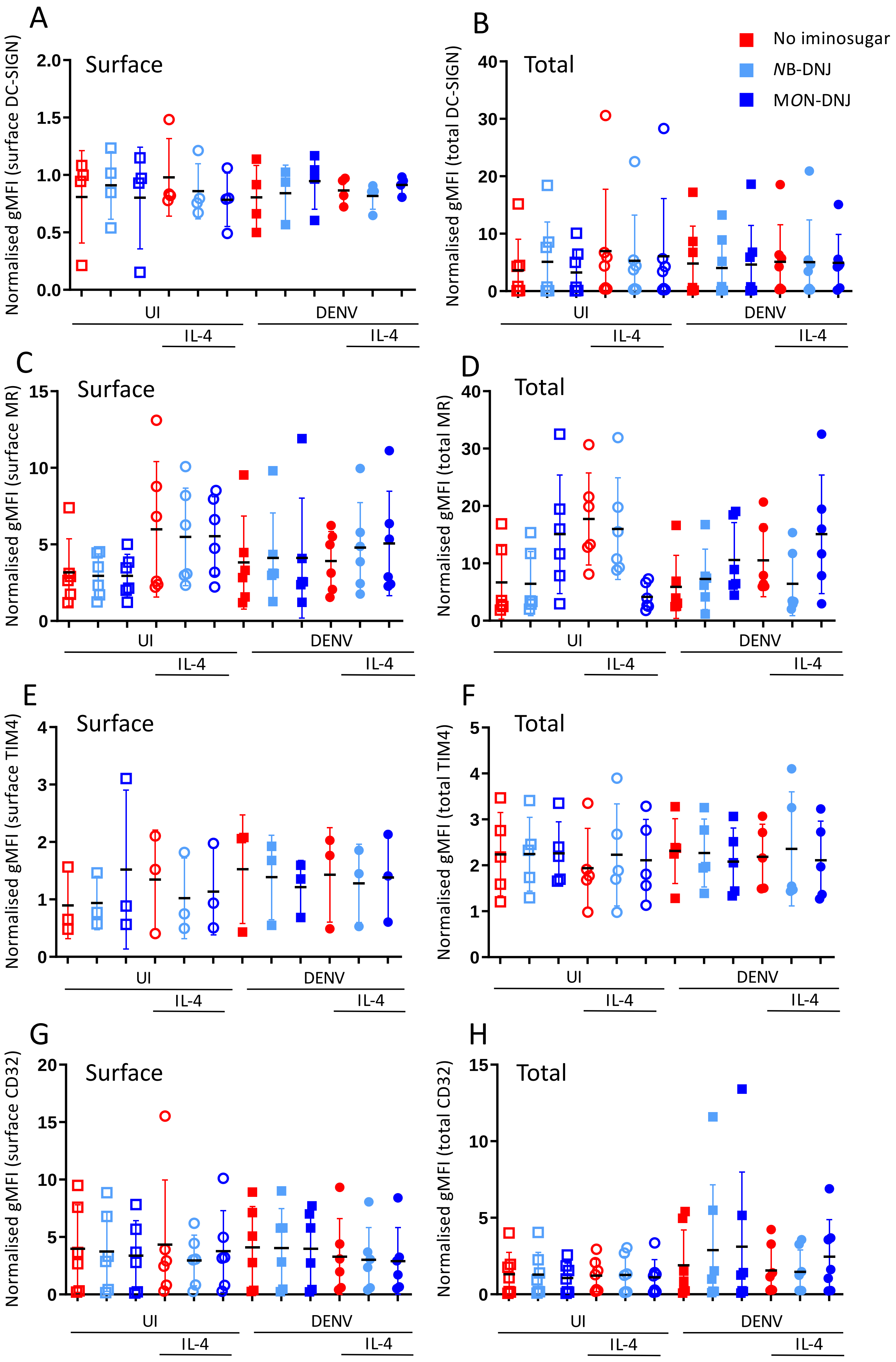
A
Surface
Normalised gMFI (surface DC-SIGN)
B
Total
Normalised gMFI (total DC-SIGN)
No iminosugar
NB-DNJ
MON-DNJ
C
Surface
Normalised gMFI (surface MR)
D
Total
Normalised gMFI (total MR)
E
Surface
Normalised gMFI (surface TIM4)
F
Total
Normalised gMFI (total TIM4)
G
Surface
Normalised gMFI (surface CD32)
H
Total
Normalised gMFI (total CD32)
UI
DENV
IL-4

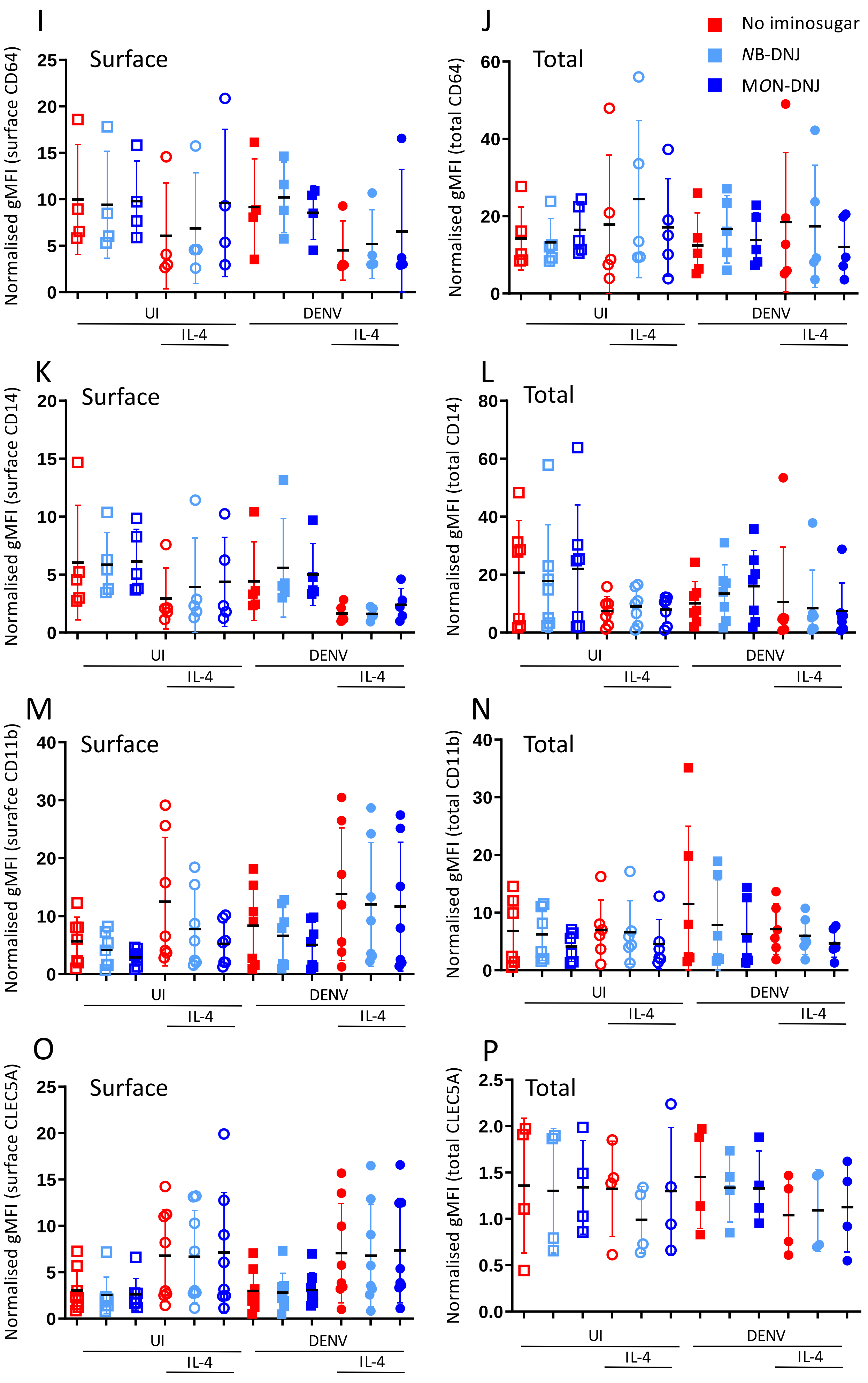

I
Surface
Normalised gMFI (surface CD64)
J
Total
Normalised gMFI (total CD64)
No iminosugar
NB-DNJ
MON-DNJ
K
Surface
Normalised gMFI (surface CD14)
L
Total
Normalised gMFI (total CD14)
M
Surface
Normalised gMFI (surafce CD11b)
N
Total
Normalised gMFI (total CD11b)
O
Surface
Normalised gMFI (surface CLEC5A)
P
Total
Normalised gMFI (total CLEC5A)
UI
DENV
IL-4

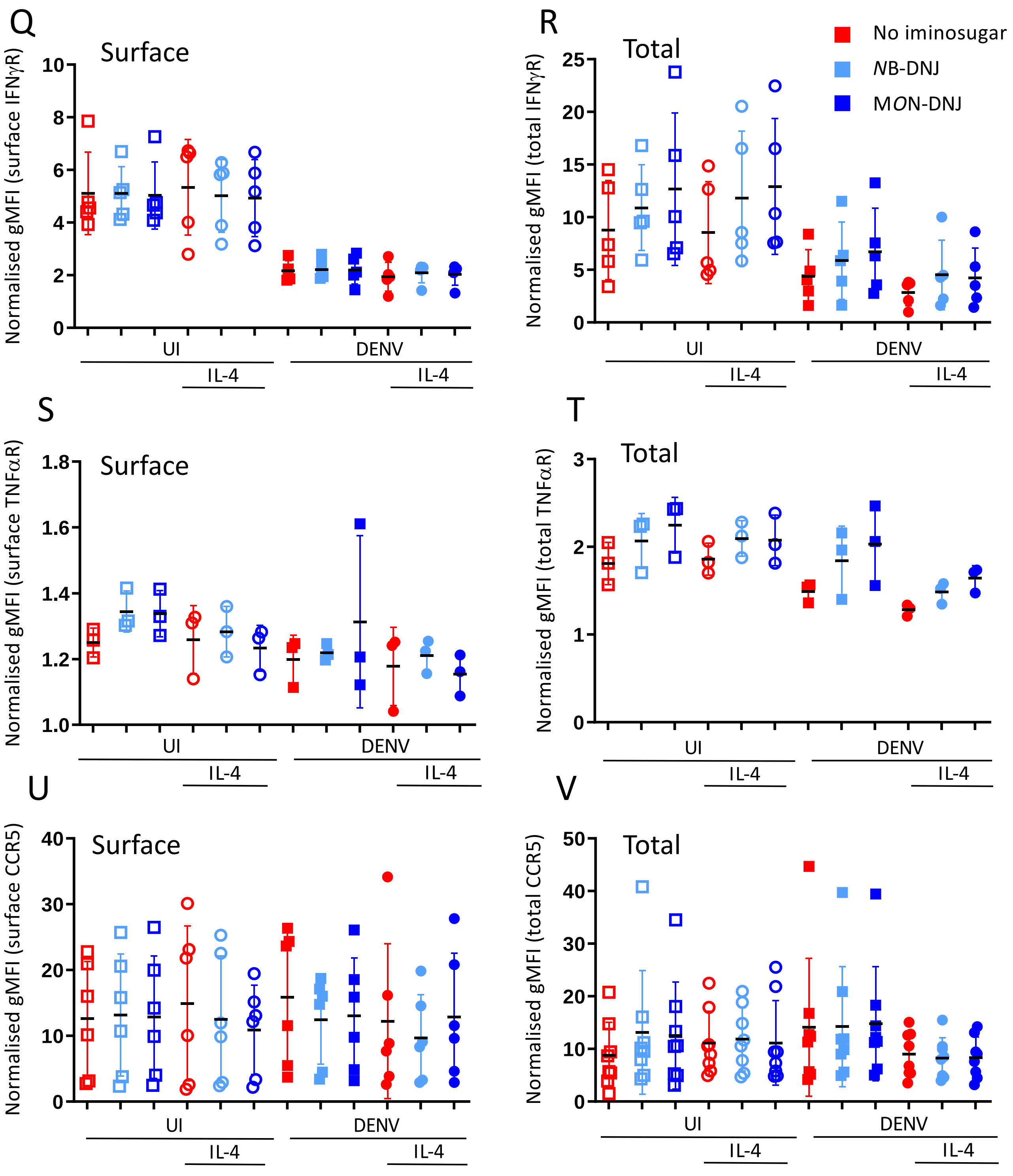

Supplemental Figure 1 legend on next page

Supplemental Figure 1: Effects of IL-4 treatment, DENV infection and iminosugar treatment on receptor expression. Monocytes isolated from the blood of multiple donors were incubated for 3 days with or without 25ng/ml rec human IL-4, before being infected with DENV2 at a MOI of 1 for 90 mins or mock-treated. Media with or without virus was aspirated from the cells and replaced with media alone, or supplemented with 100mM *N*B-DNJ or 25mM M*O*N-DNJ. Following 2 days incubation, the cells were resuspended by scraping, fixed with 1% v/v paraformaldehyde for an hour prior to blocking of non-specific binding sites with PBS containing 5mM EDTA, 5% heat-inactivated goat serum, 5% heat-inactivated rabbit serum and, for total receptor expression, 0.1% w/v saponin to permeabilise the cells. Primary antibodies anti-DC-SIGN, anti-MR, anti-TIM4, anti-CD32a, anti-CD64, anti-CD14, anti-CD11b, anti-CLEC5A, anti-IFNγR, anti-TNFαR and anti-CCR5 (Abcam, AdB Serotec, BioRad, BioLegend and R&D Systems) were used at the manufacturers' recommended concentrations. Primary antibodies were detected with Alexafluor 488- or phycoerythrin-conjugated- anti-mouse antibody (eBioscience) or FITC-conjugated anti-rabbit antibody (Invitrogen). Binding was quantified on a FACSCalibur flow cytometer and analysed using CellQuest software. Data from 5,000-10,000 events was gated to exclude debris and geometric mean fluorescent intensity (gMFI) obtained. For each experiment, cells stained with secondary antibody only and isotype controls were included. The gMFI of surface and total receptor expression was normalised to matching isotype control antibodies and is shown for DC-SIGN, MR, TIM4, CD32a, CD64, CD14, CD11b, CLEC5A, IFNγR, TNFαR and CCR5 under the twelve conditions. Normalised gMFI levels from cells from multiple donors are shown individually, as well as mean and SD.

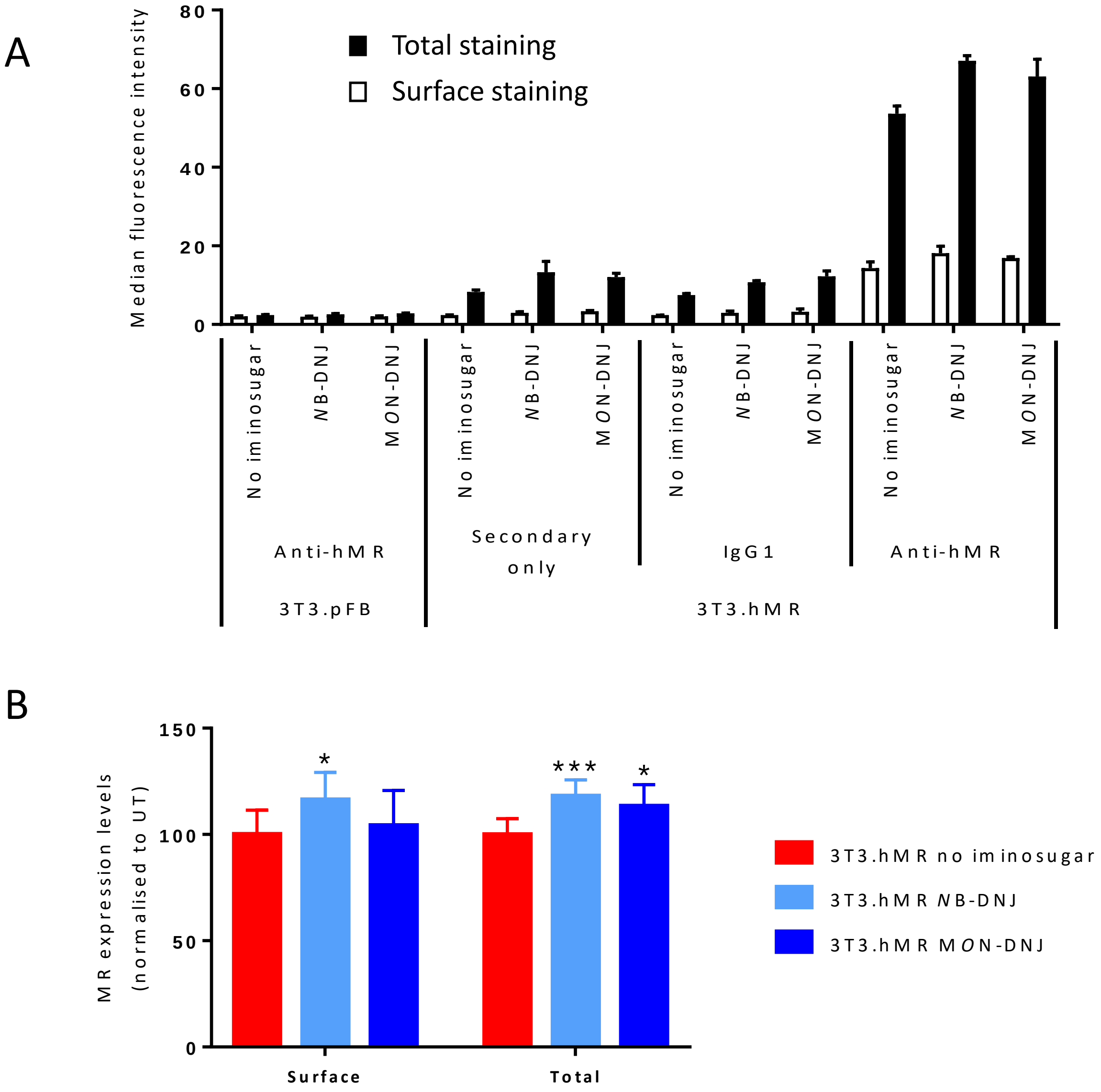

Supplemental Figure 2: Minimal effect of iminosugars on MR expression on transfected cells. 3T3.hMR transfectants were grown with 100µM *N*B-DNJ or 25µM M*O*N-DNJ for 2 days, fixed, stained with or without permeabilisation and total and surface MR expression levels, respectively, determined by flow cytometry. (A) A representative experiment shows median fluorescence intensity of total (filled bars) and surface (open bars) antibody staining specific for human MR on 3T3 cells transfected with vector alone (3T3.pFB) or with a vector containing the gene for the human MR (3T3.hMR), after cells were grown with or without iminosugars. As background and specificity controls, cells were stained without a primary antibody or with an IgG1 isotype control, respectively. Bars show mean +/- SD of staining from triplicate wells. This comparison of surface and total receptor expression demonstrates that the majority of MR was expressed intracellularly in 3T3.hMR transfectants, as has been described on primary MDMΦ (Pontow et al., 1992, Wileman et al., 1984). (B) Pooled data from three experiments, each conducted in triplicate, showing surface and total expression levels of MR on 3T3.hMR where median fluorescence intensity was normalised to that on untreated cells. * $p<0.05$, ** $p<0.005$, *** $p<0.0005$. Iminosugars slightly (1.3-fold), but significantly, upregulated surface and total expression levels on 3T3.hMR cells. Surface MR expression was significantly higher following growth in *N*B-DNJ, in comparison with 'no iminosugar' ($p = 0.0346$) as determined by one-way ANOVA. Total MR levels were significantly higher following growth in either *N*B-DNJ ($p = 0.0003$) or M*O*N-DNJ ($p = 0.0056$) in comparison with no iminosugar as determined by one-way ANOVA.

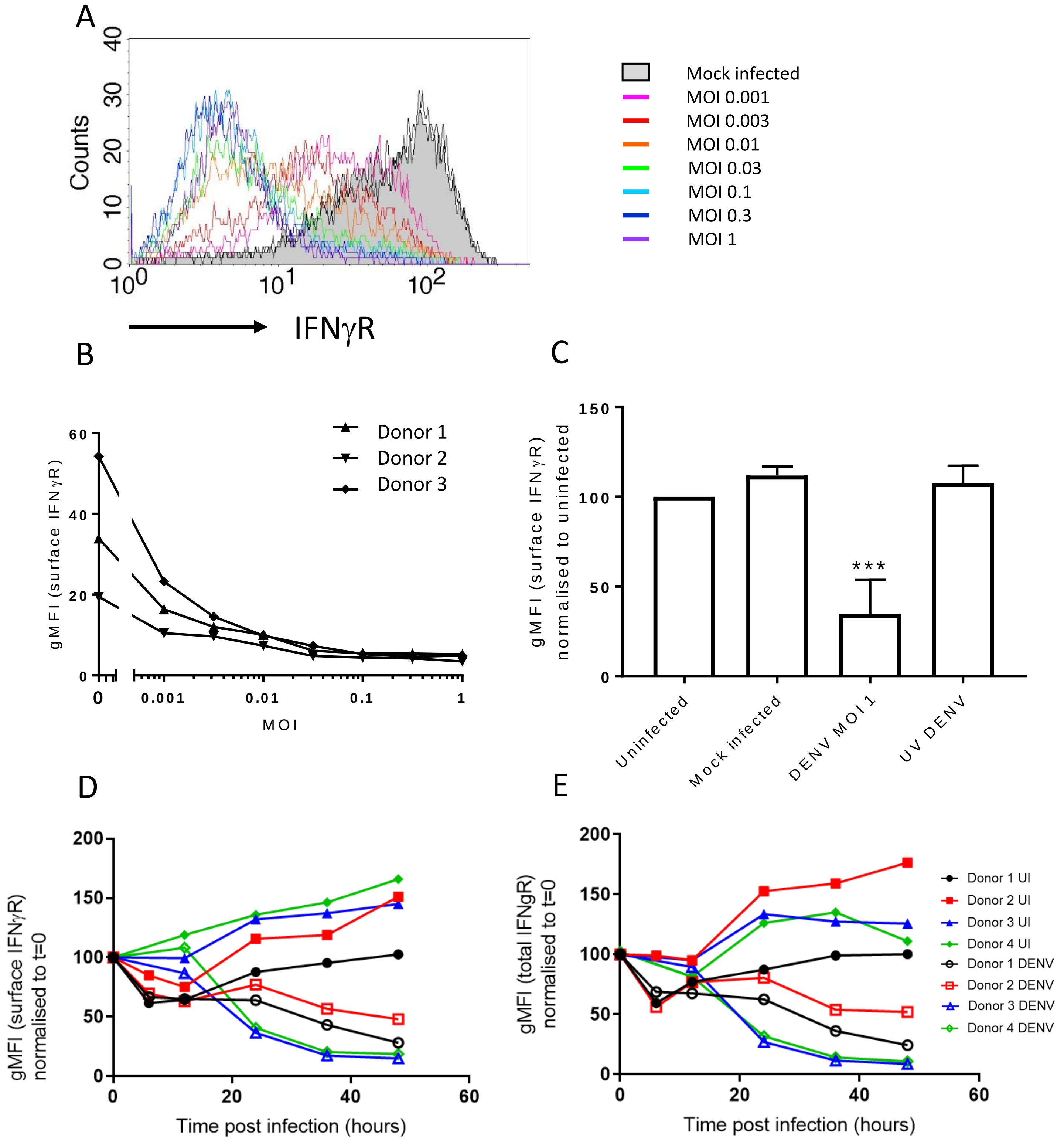

Supplemental Figure 3 legend on next page

Supplemental Figure 3: Effect of titrations of DENV infection and time on surface IFNγ receptor expression on macrophages. Monocytes isolated from the blood of multiple donors were incubated for 3 days with 25 ng/ml rec human IL-4 before being infected with DENV2 at MOI ranging from 0.001 to 1 for 90 mins, with equivalent UV-irradiated DENV2 to MOI 1, or mock-infected. UV-inactivation of DENV (UV DENV) was performed by 10 min irradiation of concentrated DENV stock 25 cm from a 254 nm lamp (UVS-18 EL Series, UVP Upland, USA). UV DENV was confirmed to have no detectable infectious DENV by plaque assay (limit of detection of 3 pfu/ml); original titres were used to calculate an equivalent MOI for treatment. Mock-infected wells received inoculum from uninfected C6/36 cell supernatant concentrated in parallel with propagation of DENV stocks. Media with or without virus was aspirated from the cells and replaced with media alone. Immediately (t = 0), or following 12, 24, 36 or 48 hours incubation (D and E), the cells were resuspended by scraping, fixed and stained as described in Supplemental Figure 1 legend and analysed by flow cytometry for receptor expression. (A) Representative histogram plot of the surface expression of IFNγR on cells from donor 1 infected with different MOI of DENV 2 days post infection. Grey filled histogram represents IFNγR expression on mock-infected cells, black line on uninfected cells, and pink to purple lines on cells infected with DENV ranging from MOI 0.001 to 1. (B) The gMFI of surface IFNγR expression on ΔMOI DENV-infected macrophages 2 days post infection on cells from 3 donors. (C) The gMFI of surface IFNγR expression normalised to that on uninfected cells on uninfected, mock-, DENV- or non-infectious UV DENV-infected macrophages 2 days post infection on cells from 4 donors, mean and SD. *** $p<0.0005$ in comparison with 'uninfected' as determined by one-way ANOVA. Time course of (D) surface or (E) total IFNγR expression post infection with DENV. The gMFI for IFNγR expression was normalised to that for t = 0. Results from mock-infected cells (incubated with uninfected C6/36-cell supernatant purified in parallel to DENV-infected supernatant from C6/36 cells) mirrored uninfected cells (data not shown).

Bibliography

Pontow, S.E., Kery, V., Stahl, P.D., 1992. Mannose receptor. Int Rev Cytol 137B, 221-244.
Wileman, T., Boshans, R.L., Schlesinger, P., Stahl, P., 1984. Monensin inhibits recycling of macrophage mannose-glycoprotein receptors and ligand delivery to lysosomes. Biochem J 220, 665-675.
