# Supplementary material for: Iminosugars counteract the downregulation of the interferon γ receptor by dengue virus
Source: Antiviral Res. 2019 Oct;170:104551. doi: 10.1016/j.antiviral.2019.104551 (PMC6891261; doi:10.1016/j.antiviral.2019.104551)
Supplement: Supplemental Table 1 [file mmc2.pdf]

Supplemental Table 1: Relevance of receptors examined in this study in the life cycle of DENV in humans.

	Receptor	Alternate names (gene name)	Role in the DENV lifecycle	References
Attachment/entry receptors	DC-SIGN	dendritic cell-specific intercellular adhesion molecule-3-grabbing non-integrin, CLEC4L (CD209)	• C-type lectin receptor that binds DENV E protein N-linked glycans for attachment to cells<br>• N-linked glycosylation on the neck domain may regulate oligomeric state<br>• Promoter single nucleotide polymorphisms (SNPs) are associated with disease severity	(Tassaneetrithep, Burgess et al. 2003, Despres, Sakuntabhai et al. 2005, Serrano-Gomez, Martinez-Nunez et al. 2007)
	MR	mannose receptor, CD206 (MRC1)	• C-type lectin receptor that binds glycans on DENV E protein for attachment to cells<br>• Glycosylation modulates binding activity<br>• Co-ordinates with CLEC5A for cooperative interaction leading to infection	(Su, Tsang et al. 2005, Miller, de Wet et al. 2008, Lo, Liou et al. 2016)
	TIM4	T-cell immunoglobulin- and mucin-domain-containing molecule (TIMD4)	• A phosphatidylserine receptor that mediates DENV entry	(Meertens, Carnec et al. 2012)
	FcγRIIA	CD32a (FCGR2A)	• Major Fc receptor implicated in antibody dependent enhancement (ADE) infection with DENV<br>• Low affinity IgG receptor that is N-glycosylated<br>• Association between SNPs in this receptor and the severity of DENV infection	(Kontny, Kurane et al. 1988, Littaua, Kurane et al. 1990, Mady, Erbe et al. 1991, Loke, Bethell et al. 2002, Rodrigo, Jin et al. 2006, Rodrigo, Block et al. 2009, Garcia, Sierra et al. 2010, Boonnak, Slike et al. 2013)
	FcγRIA	CD64 (FCGR1A)	• N-linked glycoprotein with high affinity for IgG antibodies that mediates phagocytosis thereby enhancing infection of DENV via immune complexes (i.e. mediates ADE)	
	CD14	(CD14)	• LPS-binding CD14-associated molecule is a receptor for DENV on human monocytes	(Chen, Wang et al. 1999, Alhoot, Wang et al. 2011)
	CD11b	Intergrin αM chain, Mac1α, CR3 α chain (ITGAM)	• Associates with CD18 to form the complement receptor 3<br>• Dengue fever patients have reduced CD11b expression on monocytes<br>• Blocking CD11b reduces DENV infection of *ex vivo* monocytes and secreted TNFα and IFNα	(Marinho, Azeredo et al. 2014)
Immune receptors	CLEC5A	MDL-1 (CLEC5A)	• C-type lectin receptor that binds DENV and in conjunction with DAP12 signals for induction of proinflammatory cytokines<br>• DENV triggering of CLEC5A activation mediates production of nitric oxide and TNFα leading to shock in mice<br>• SNPs in CLEC5A are associated with dengue disease severity	(Chen, Lin et al. 2008, Cheung, Shen et al. 2011, Xavier-Carvalho, Gibson et al. 2013, Xavier-Carvalho, Cezar et al. 2017)
	CCR5	C-C chemokine receptor 5, CD195 (CCR5)	• Inflammatory chemokine receptor for multiple chemokines, including MIP-1α, MIP-1β and RANTES<br>• DENV infection induces release of MIP-1β and RANTES<br>• CCR5 activation is required for DENV replication	(Marques, Guabiraba et al. 2015)
	IFNγR1	CD119 (IFNGR1)	• Ligand binding chain of the IFNγ receptor that mediates the response to IFNγ, an important cytokine produced during dengue infection that directs innate and adaptive immune responses	(Diamond, Edgil et al. 2000)
	TNFαR1	CD120a (TNFRSF1A)	• Main receptor for soluble TNFα, binding promotes production of pro-inflammatory cytokines	(Santos, de Moura et al. 2017)

Alhoot, M. A., S. M. Wang and S. D. Sekaran (2011). "Inhibition of dengue virus entry and multiplication into monocytes using RNA interference." PLoS Negl Trop Dis **5**(11): e1410.
Boonnak, K., B. M. Slike, G. C. Donofrio and M. A. Marovich (2013). "Human FcgammaRII cytoplasmic domains differentially influence antibody-mediated dengue virus infection." J Immunol **190**(11): 5659-5665.
Chen, S. T., Y. L. Lin, M. T. Huang, M. F. Wu, S. C. Cheng, H. Y. Lei, C. K. Lee, T. W. Chiou, C. H. Wong and S. L. Hsieh (2008). "CLEC5A is critical for dengue-virus-induced lethal disease." Nature **453**(7195): 672-676.
Chen, Y. C., S. Y. Wang and C. C. King (1999). "Bacterial lipopolysaccharide inhibits dengue virus infection of primary human monocytes/macrophages by blockade of virus entry via a CD14-dependent mechanism." J Virol **73**(4): 2650-2657.
Cheung, R., F. Shen, J. H. Phillips, M. J. McGeachy, D. J. Cua, P. G. Heyworth and R. H. Pierce (2011). "Activation of MDL-1 (CLEC5A) on immature myeloid cells triggers lethal shock in mice." J Clin Invest **121**(11): 4446-4461.
Despres, P., A. Sakuntabhai and C. Julier (2005). "[A variant in the CD209 (DC-SIGN) promoter is associated with severity of dengue disease]." Med Sci (Paris) **21**(11): 905-906.
Diamond, M. S., D. Edgil, T. G. Roberts, B. Lu and E. Harris (2000). "Infection of human cells by dengue virus is modulated by different cell types and viral strains." J Virol **74**(17): 7814-7823.
Garcia, G., B. Sierra, A. B. Perez, E. Aguirre, I. Rosado, N. Gonzalez, A. Izquierdo, M. Pupo, D. R. Danay Diaz, L. Sanchez, B. Marcheco, K. Hirayama and M. G. Guzman (2010). "Asymptomatic dengue infection in a Cuban population confirms the protective role of the RR variant of the FcgammaRIIa polymorphism." Am J Trop Med Hyg **82**(6): 1153-1156.
Kontny, U., I. Kurane and F. A. Ennis (1988). "Gamma interferon augments Fc gamma receptor-mediated dengue virus infection of human monocytic cells." J Virol **62**(11): 3928-3933.
Littaua, R., I. Kurane and F. A. Ennis (1990). "Human IgG Fc receptor II mediates antibody-dependent enhancement of dengue virus infection." J Immunol **144**(8): 3183-3186.
Lo, Y. L., G. G. Liou, J. H. Lyu, M. Hsiao, T. L. Hsu and C. H. Wong (2016). "Dengue Virus Infection Is through a Cooperative Interaction between a Mannose Receptor and CLEC5A on Macrophage as a Multivalent Hetero-Complex." PLoS One **11**(11): e0166474.
Loke, H., D. Bethell, C. X. Phuong, N. Day, N. White, J. Farrar and A. Hill (2002). "Susceptibility to dengue hemorrhagic fever in vietnam: evidence of an association with variation in the vitamin d receptor and Fc gamma receptor IIa genes." Am J Trop Med Hyg **67**(1): 102-106.
Mady, B. J., D. V. Erbe, I. Kurane, M. W. Fanger and F. A. Ennis (1991). "Antibody-dependent enhancement of dengue virus infection mediated by bispecific antibodies against cell surface molecules other than Fc gamma receptors." J Immunol **147**(9): 3139-3144.
Marinho, C. F., E. L. Azeredo, A. Torrentes-Carvalho, A. Marins-Dos-Santos, C. F. Kubelka, L. J. de Souza, R. V. Cunha and L. M. de-Oliveira-Pinto (2014). "Down-regulation of complement receptors on the surface of host monocyte even as in vitro complement pathway blocking interferes in dengue infection." PLoS One **9**(7): e102014.
Marques, R. E., R. Guabiraba, J. L. Del Sarto, R. F. Rocha, A. L. Queiroz, D. Cisalpino, P. E. Marques, C. C. Pacca, C. T. Fagundes, G. B. Menezes, M. L. Nogueira, D. G. Souza and M. M. Teixeira (2015). "Dengue virus requires the CC-chemokine receptor CCR5 for replication and infection development." Immunology **145**(4): 583-596.
Meertens, L., X. Carnec, M. P. Lecoin, R. Ramdasi, F. Guivel-Benhassine, E. Lew, G. Lemke, O. Schwartz and A. Amara (2012). "The TIM and TAM families of phosphatidylserine receptors mediate dengue virus entry." Cell Host Microbe **12**(4): 544-557.

Miller, J. L., B. J. de Wet, L. Martinez-Pomares, C. M. Radcliffe, R. A. Dwek, P. M. Rudd and S. Gordon (2008). "The mannose receptor mediates dengue virus infection of macrophages." PLoS Pathog **4**(2): e17.

Rodrigo, W. W., O. K. Block, C. Lane, S. Sukupolvi-Petty, A. P. Goncalvez, S. Johnson, M. S. Diamond, C. J. Lai, R. C. Rose, X. Jin and J. J. Schlesinger (2009). "Dengue virus neutralization is modulated by IgG antibody subclass and Fcgamma receptor subtype." Virology **394**(2): 175-182.

Rodrigo, W. W., X. Jin, S. D. Blackley, R. C. Rose and J. J. Schlesinger (2006). "Differential enhancement of dengue virus immune complex infectivity mediated by signaling-competent and signaling-incompetent human Fcgamma RIA (CD64) or FcgammaRIIA (CD32)." J Virol **80**(20): 10128-10138.

Santos, A., E. L. de Moura, J. M. Ferreira, A. W. A. de Moura, A. D. S. Ferreira, R. P. Bezerra, D. de Siqueira Figueiredo, K. F. de Farias, T. G. de Andrade and E. V. M. de Souza Figueiredo (2017). "Association of TNFA (-308G/A), IFNG (+874 A/T) and IL-10 (-819 C/T) polymorphisms with protection and susceptibility to dengue in Brazilian population." Asian Pac J Trop Med **10**(11): 1065-1071.

Serrano-Gomez, D., R. T. Martinez-Nunez, E. Sierra-Filardi, N. Izquierdo, M. Colmenares, J. Pla, L. Rivas, J. Martinez-Picado, J. Jimenez-Barbero, J. L. Alonso-Lebrero, S. Gonzalez and A. L. Corbi (2007). "AM3 modulates dendritic cell pathogen recognition capabilities by targeting DC-SIGN." Antimicrob Agents Chemother **51**(7): 2313-2323.

Su, Y. R., C. Tsang, T. Bakker, J. Harris, S. Gordon, R. A. Dwek, L. Martinez-Pomares and P. M. Rudd (2005). "Glycosylation influences the ligand binding activities of mannose receptor." Adv Exp Med Biol **564**: 25-26.

Tassaneetrithep, B., T. H. Burgess, A. Granelli-Piperno, C. Trumpfheller, J. Finke, W. Sun, M. A. Eller, K. Pattanapanyasat, S. Sarasombath, D. L. Birx, R. M. Steinman, S. Schlesinger and M. A. Marovich (2003). "DC-SIGN (CD209) mediates dengue virus infection of human dendritic cells." J Exp Med **197**(7): 823-829.

Xavier-Carvalho, C., R. Cezar, N. M. Freire, C. M. M. Vasconcelos, V. E. F. Solorzano, T. G. de Toledo-Pinto, L. G. Fialho, R. F. do Carmo, L. R. S. Vasconcelos, M. T. Cordeiro, P. Baptista, E. L. de Azeredo, R. V. da Cunha, L. J. de Souza, A. G. Pacheco, C. F. Kubelka, P. Moura and M. O. Moraes (2017). "Association of rs1285933 single nucleotide polymorphism in CLEC5A gene with dengue severity and its functional effects." Hum Immunol **78**(10): 649-656.

Xavier-Carvalho, C., G. Gibson, P. Brasil, R. X. Ferreira, R. de Souza Santos, O. Goncalves Cruz, S. A. de Oliveira, M. de Sa Carvalho, A. G. Pacheco, C. F. Kubelka and M. O. Moraes (2013). "Single nucleotide polymorphisms in candidate genes and dengue severity in children: a case-control, functional and meta-analysis study." Infect Genet Evol **20**: 197-205.
