# Supplementary material for: Iminosugars counteract the downregulation of the interferon γ receptor by dengue virus
Source: Antiviral Res. 2019 Oct;170:104551. doi: 10.1016/j.antiviral.2019.104551 (PMC6891261; doi:10.1016/j.antiviral.2019.104551)
Supplement: Supplemental Tables 2 and 3 [file mmc3.pdf]

Supplemental Tables 2 and 3: Significance of changes in <u>total</u> (Table 2) and <u>surface</u> (Table 3) receptor expression following 3 days with or without IL-4-treatment, followed by infection with DENV at a MOI 1, then 2 days incubation with or without 100μM *N*B-DNJ or 25μM M*O*N-DNJ. Receptor expression was assessed by flow cytometry on permeabilised cells (Table 2) and non-permeabilised cells (Table 3) with primary antibodies as listed, followed by a PE-labelled secondary antibody and quantified by gMFI, which was normalised to isotype control staining. Data from flow cytometry experiments were analysed via three-way ANOVA using SigmaPlot® 13 (Systat Software Inc.) and were considered statistically significant if $p < 0.05$. Briefly, IL-4 treatment, DENV infection and iminosugar treatment were used to detect significant differences between each category. Post-hoc pairwise multiple comparisons were performed using the Holm-Šídák method. Arrows indicate whether expression was up- or down-regulated.

Table 2: Significance of changes in <u>total</u> receptor expression

	Antibody specificity	p value (direction of regulation)			
		**IL-4**	**DENV**	***N*B-DNJ**	**M*O*N-DNJ**
Attachment/entry receptors	DC-SIGN (CD209)	0.384	0.862	0.898	0.836
	MR (CD206)	<0.001 (↑)	0.003 (↓)	0.924	0.606
	TIM4	0.727	0.825	0.718	0.694
	FcγRIIA(CD32a)	0.430	0.051	0.788	0.374
	FcγRIA (CD64)	0.300	0.529	0.627	0.817
	CD14	0.007 (↓)	0.291	0.989	0.758
	CD11b	0.424	0.347	0.475	0.090
Immune receptors	CLEC5A (MDL-1)	0.177	0.793	0.549	0.915
	CCR5	0.094	0.982	0.607	0.655
	IFNγR (CD119)	0.538	<0.001 (↓)	0.110	0.050 (↑)
	TNFαR (CD120a)	0.057	<0.001 (↓)	0.013 (↑)	0.002 (↑)

Table 3: Significance of changes in <u>surface</u> receptor expression.

	Antibody specificity	p value (direction of regulation)			
		**IL-4**	**DENV**	***N*B-DNJ**	**M*O*N-DNJ**
Attachment/entry receptors	DC-SIGN (CD209)	0.433	0.763	0.742	0.742
	MR (CD206)	0.023 (↑)	0.941	0.891	0.820
	TIM4	0.943	0.408	0.640	0.970
	FcγRIIA (CD32a)	0.558	0.867	0.882	0.955
	FcγRIA (CD64)	0.053	0.412	0.783	0.537
	CD14	0.002 (↓)	0.085	0.643	0.468
	CD11b	0.003 (↑)	0.051	0.256	0.064
Immune receptors	CLEC5A (MDL-1)	<0.001 (↑)	0.794	0.794	0.944
	CCR5	0.601	0.948	0.477	0.599
	IFNγR (CD119)	0.769	<0.001 (↓)	0.928	0.803
	TNFαR (CD120a)	0.106	0.046 (↓)	0.179	0.447
